# An association between crypt apoptotic bodies and mucosal flattening in celiac disease patients exposed to dietary gluten

**DOI:** 10.1186/s13000-019-0878-1

**Published:** 2019-08-31

**Authors:** Michael Lee, Shane Betman, Alina Iuga, Hui-Min Yang, Jude Fleming, Peter H. R. Green, Benjamin Lebwohl, Stephen M. Lagana

**Affiliations:** 1 Department of Pathology and Cell Biology, Columbia University Medical Center, 630 West 168th Street, VC14-240A, New York, NY 10032 USA; 2 Internal Medicine, Columbia University Medical Center, New York, NY USA; 3 Celiac Disease Center, Columbia University Medical Center, New York, NY USA

**Keywords:** Pathology, Celiac disease, Apoptosis

## Abstract

**Background:**

Celiac disease (CD) is characterized histologically by inflammation and villous atrophy. Villous atrophy is thought to result from a disruption of epithelial cellular proliferation and death. Epithelial cells in intestinal mucosa normally proliferate in the crypts and migrate towards the lumen, eventually dying. Apoptotic bodies in crypts are usually abnormal and are associated with certain disease states. The presence of crypt apoptosis in celiac disease has not been thoroughly examined by routine histologic assessment of crypt apoptotic body count (ABC).

**Methods:**

We quantified the ABC in duodenal biopsies from celiac patients before and after initiation of a gluten-free diet (GFD). We examined twenty-three duodenal biopsies from adult patients with celiac disease at diagnosis and following GFD and determined the maximum ABC in 10 consecutive crypts. Fourteen biopsies from heartburn patients served as controls.

**Results:**

Mean duration between paired biopsies was 2.9 (0.5–8.5) years. Mean maximum ABC in active celiac disease was 5.44 per crypt and decreased to 2.60 with GFD (*p* = <.0001). The mean maximum ABC in controls was 1.79, lower than both active celiac disease and GFD (*p* = <.0001 and *p* = .019 respectively). Flat lesions with total villous atrophy (mean: 6.44) showed a higher ABC compared to non-flat lesions (mean: 4.87); *p* = .04.

**Conclusions:**

Crypt ABC is markedly elevated in active celiac disease and decreases significantly with GFD, however it does not achieve normalcy. Total villous atrophy is associated with a higher ABC than all other lesions. Crypt apoptosis is likely a significant contributor to villous atrophy in celiac disease and can be appreciated by routine histologic examination.

## Background

Celiac disease (CD) is an autoimmune inflammatory disorder caused by the consumption of gluten, a dietary protein, in genetically susceptible individuals. Some of the digestion products are resistant to further degradation and induce a cascade of inflammatory responses including recruitment and activation of intraepithelial lymphocytes, antigen presenting cells and a release of cytokines. These inflammatory responses cause tissue damage and subsequent villous atrophy [1, 2].

The prevalence of CD in the United States is estimated to be nearly 1%, though many cases are thought to be undiagnosed [3]. The clinical manifestations are broad, including diarrhea, constipation, weight loss, vomiting, osteoporosis, dermatitis herpetiformis, infertility, and neuropsychiatric issues [4]. CD has also been linked to a 2 to 4-fold increased risk of lymphoma, oral, esophageal, small intestinal, and hepatocellular carcinoma, and a 1.4-fold increase in overall mortality [5, 6]. Treatment requires commitment to a life-long gluten-free diet (GFD).

The diagnosis of CD is multidisciplinary, and is established by a characteristic duodenal biopsy, usually in a patient with positive serologic markers, and improvement of clinical symptoms after initiation of a GFD [7]. Genetic testing is useful in borderline presentations. The characteristic histologic findings in active celiac disease are intraepithelial lymphocytosis (IEL), crypt hyperplasia, and villous atrophy [8]. Upon initiation of a GFD, symptoms typically improve within weeks and serologic markers can normalize within months [4], however villous height has been shown to take a median of 3.8 years to recover [9]. In some patients, villous height recovers, but intraepithelial lymphocytosis persists, the implications of which are unclear [10]. In other patients, symptoms and villous height do not recover despite adherence to a GFD. This is termed refractory celiac disease (RCD) and is further sub-classified into RCD type I with a normal IEL phenotype, and RCD type II, with an abnormal, clonal IEL phenotype, although the diagnosis is complicated by clinically insignificant subclones [11, 12].

The mechanism of villous atrophy is thought to be a disruption in the balance between epithelial cell proliferation and death [13]. Intestinal epithelial cells normally proliferate in crypts and migrate towards the lumen where they function for a period of time and subsequently undergo apoptosis and are shed. The normal number of crypt apoptotic bodies in small intestinal mucosa is controversial, though for the purposes of graft-versus-host-disease diagnosis, the National Institutes of Health consensus statements suggests 1 per biopsy fragment [14]. This value is highly sensitive, though not specific for graft-versus-host disease [15, 16].

Despite this confusion about the threshold, excessive crypt apoptosis is abnormal and is associated with certain disease states, such as graft-versus-host disease, acute cellular rejection in small bowel allografts, and medication injury, such as in patients with toxicity due to mycophenolate mofetil [16, 17]. Apoptosis in intestinal crypts has been demonstrated in biopsies of active celiac disease by a variety of methods, including nick-end labelling assays [15, 17] and immunohistochemistry for epithelial apoptosis markers [18–20]. Studies have shown an increase in the degradation end products of apoptosis (i.e. M30, H2AX, cleaved caspase 3), down regulation of apoptotic inhibitor Bcl2, and upregulation of Ki-67 proliferation index in celiac disease [19]. This evidence is also corroborated by studies showing in vitro induction of enterocyte apoptosis via exposure to wheat gliadin [21].

Although crypt apoptosis has been observed in CD with ancillary techniques, a histologic assessment of crypt apoptotic body count (ABC) in celiac disease has not been reported. Histologic assessment of ABC is the mainstay of diagnosis for the aforementioned conditions associated with increased apoptosis, and ancillary techniques have not become part of clinical practice. The intent of this study is to assess and quantify crypt apoptosis as seen on routine hematoxylin and eosin stained sections of biopsies in active celiac disease, and to correlate ABC with the degree of villous blunting and atrophy.

## Methods

Following approval of the Columbia University Institutional Review Board, we queried the electronic health record of patients attending the Celiac Disease Center at Columbia University Medical Center and identified twenty-three adult patients with a new diagnosis of CD who had both a biopsy with active celiac disease and a follow-up biopsy after initiation of a GFD. Fourteen patients without CD who underwent duodenal biopsy at the same medical center for evaluation of suspected acid reflux served as controls. The biopsies were from the second part of the duodenum and examined by three experienced gastrointestinal pathologists (ML, HY and SL). One assigned a Marsh grade (SL) according to the modified Marsh-Oberhuber classification [22] and all recorded the maximum number of apoptotic bodies in ten consecutive crypts. Discrepancies were resolved by averaging results. This value was designated as the apoptotic body count. The pathologists were blinded with respect to the medical history of all examined cases.

**Fig. 1.**
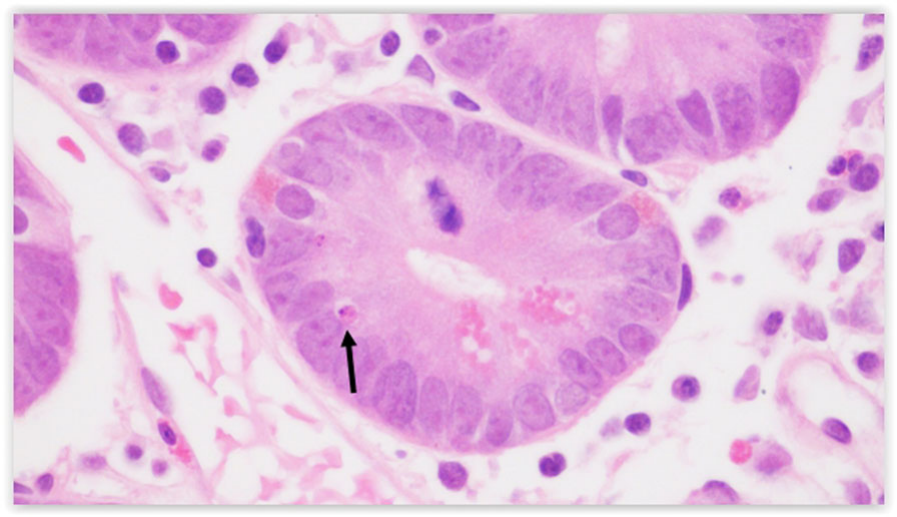
An apoptotic body demonstrates condensed nuclear chromatin and eosinophilia (arrow)

**Fig. 2.**
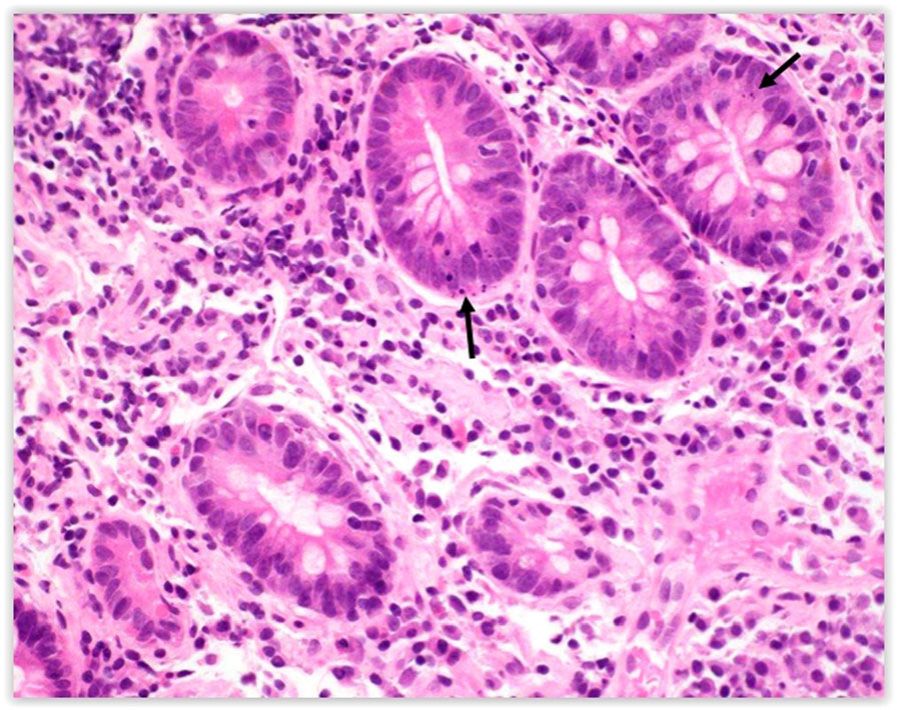
Multiple crypt apoptotic bodies and fragments of karyorrhectic debris (arrows) fulfill criteria for an apoptotic body in a patient with a Marsh 3C lesion

We used the German-Austrian-Swiss Consortium “liberal” criteria for an apoptotic body, defined as either condensed nuclear chromatin with eosinophilic cytoplasm or at least two fragments of nuclear, karyorrhectic debris with clearing and vacuolization, Figures 1 and 2 [23, 24]. The ABCs of the active celiac disease and post-treatment biopsies were compared with a paired t-test. Welch’s t-test was used to compare the study biopsies to control biopsies and to compare completely flat (Marsh 3C, corresponding to total villous atrophy) to non-flat lesions. All statistical tests were two-sided.

## Results

There were 23 patients with a mean age of 44 years at the time of initial biopsy. This group included 9 males and 14 females. The mean interval from initial diagnostic biopsy to post-treatment biopsy was 3.02 years and the mean was 2.9 years (range: 0.5–8.5 years). The most common reason for upper gastrointestinal tract biopsies was abdominal pain or dyspepsia. The mean tissue transglutaminase was 4.8 times the upper limit of normal at diagnostic biopsy and 0.7 times the upper limit of normal at follow-up biopsy. Two active celiac disease biopsies and five GFD biopsies were not assigned Marsh grades due to suboptimal tissue orientation precluding accurate assessment of villous architecture (in the two active celiac disease cases, the diagnoses were based on the presence of inflammation, particularly intraepithelial lymphocytosis, in the context of elevated CD serologies).

**Fig. 3.**
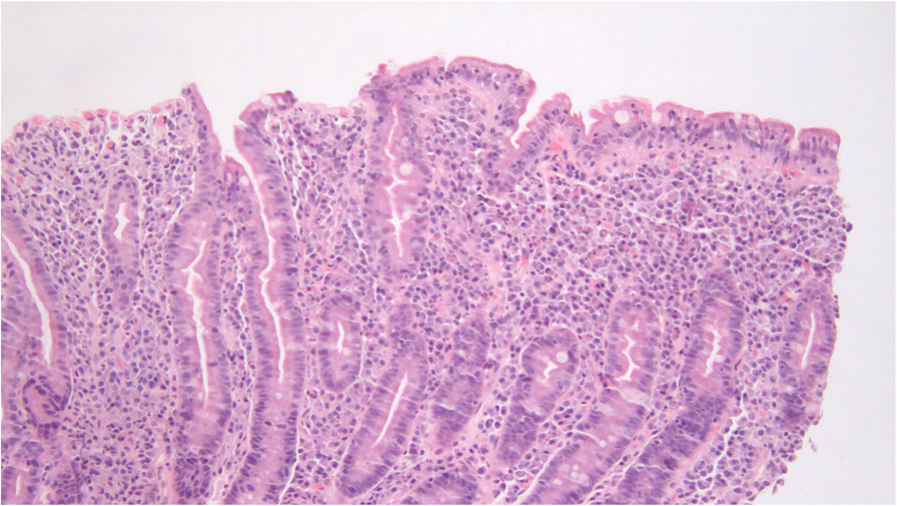
This flat lesion returned to a state of relative normalcy after 24 months. Such recovery was associated with a reduction of the ABC, though it remained higher than the mean ABC of controls

**Fig. 4.**
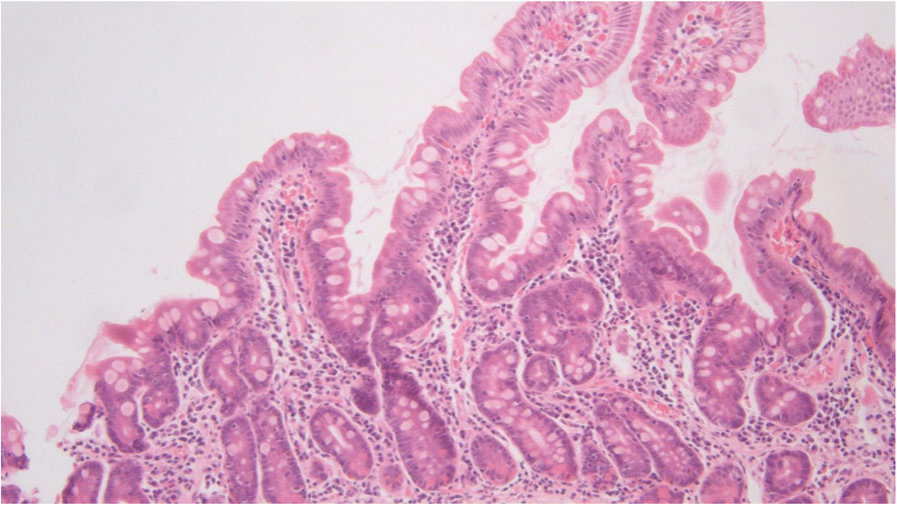
The flat lesion from Figure 3 returned to a state of normalcy after 24 months

The active celiac disease biopsies consisted of 18 patients (78%) with villous atrophy (Marsh 3 lesions), 9 of which showed complete villous atrophy (50% of Marsh 3 cases, 39% of total cases). Among the post-treatment biopsies, 5 patients (21%) had persistent villous atrophy with intraepithelial lymphocytosis (Marsh 3 lesion); these patients had an average interval between biopsies of 2.61 (0.98–3.31) years, Figures 3 and 4. Fifteen of the post-treatment biopsies showed normal villous architecture. All control biopsy cases from patients with dyspepsia did not show any degree of villous atrophy.

The mean maximum ABC among the active celiac disease biopsies was 5.44, significantly higher than the post-treatment biopsy mean of 2.60 (*p* = <.0001), see Table 1. The mean maximum ABC in controls was 1.79, less than both active and treated CD (*p* = <.0001 and *p* = .015 respectively). Completely flat lesions had a mean maximum ABC of 6.44, which was higher than the mean of 4.88 for non-flat lesions (*p* = .04). In the group of follow-up biopsies, the mean maximum ABC was 3.40 in patients with persistent villous atrophy and 2.55 in patients without (*p* = .023). The degree of villous atrophy was improved in each of the 5 patients (3 went from total to partial, 1 from total to subtotal, and 1 subtotal to partial).

**Table 1:** Mean maximum crypt ABC when comparing active celiac disease biopsies with post gluten-free diet biopsies

	Mean Maximum Crypt Apoptotic Body Count (ABC)
Controls	1.79 (*n* = 14)
All active celiac disease (ACD)	5.44 (*n* = 23)
ACD – Marsh 1	4.00 (n = 2)
ACD – Marsh 2	5.00 (n = 1)
ACD – Marsh 3A	4.67 (*n* = 6)
ACD – Marsh 3B	6.66 (*n* = 3)
ACD – Marsh 3C	6.44 (*n* = 9)
Post gluten free diet	2.60 (n = 23)

## Discussion

In this study, crypt ABC was significantly higher in active celiac disease compared to the GFD and control groups. After treatment on a gluten-free diet, crypt ABC decreased although it did not achieve normalcy. There was a direct correlation between crypt ABC and the degree of villous atrophy in the active celiac disease group. Patients with complete atrophy of villi (Marsh 3C) had higher ABCs than all other Marsh lesions (1, 2, 3A or 3B). These results suggest that the capacity for cellular regeneration is outpaced by the degree of apoptosis, including crypt apoptosis, highlighting the importance of epithelial apoptosis in the pathobiology of villous atrophy. Indeed, increased crypt apoptosis is seen in various conditions which are associated with villous atrophy, including olmesartan-associated enteropathy and autoimmune enteropathy [25, 26]. The patients who were reportedly gluten-free, but had ongoing inflammation and villous atrophy, may have these abnormalities for a number of reasons. These patients were not clinically thought to be refractory, and may have been inadvertently exposed to gluten, causing persistent villous atrophy. Alternatively, they may have been slow to heal despite being strictly adherent; prior studies suggest that increased time on the gluten-free diet is predictive of healing [9, 27]. The observation that this group had a higher ABC than the GFD patients without inflammation and villous atrophy is unsurprising. We consider it more notable that a statistically significant reduction in ABC compared to their initial biopsies accompanied mild improvement in villous architecture. This suggests a tight correlation between ABC and villous atrophy.

Practically, searching for crypt apoptotic bodies is a time-consuming endeavor requiring diligent examination of multiple levels at high power magnification. A lymphocyte can resemble karyorrhectic debris and artefactual vacuolation can mimic the cytoplasmic clearing of an apoptotic body. Small bowel crypt apoptosis is also seen in other diseases such as graft-versus-host disease, acute cellular rejection in small bowel allografts, and medication toxicity. An examination of the patient’s medical record could elucidate laboratory studies for serologic markers of celiac disease, the presence of a small bowel transplant operation and data regarding exposure to a medication from the ever-growing list of apoptosis inducing pharmacologic agents (e.g. mycophenolate mofetil, methotrexate and tumor necrosis factor alpha inhibitors) [28].

There are also numerous conditions that demonstrate intraepithelial lymphocytosis and/or villous atrophy, histologic features that overlap with celiac disease. This differential includes duodenal injury associated with *H. pylori* gastritis, autoimmune enteropathy, tropical sprue, common variable immunodeficiency, medication effects (particularly angiotensin receptor blockers), inflammatory bowel disease, and microvillus inclusion disease [29–32]. Understanding the full spectrum of histopathologic features in celiac disease and other entities on the differential is vital in resolving this differential.

## Conclusion

Assessing and quantifying ABCs by routine light microscopy and tracking changes in successive biopsies may be a useful histologic criterion to assess response to GFD. Additional studies are needed to determine the predictive value of ABC count on villous recovery and whether it warrants discussion as a useful prognostic marker. Also, given the relative commonality of celiac disease, it is useful to keep this feature in mind when evaluating intestinal biopsies for the more esoteric conditions associated with crypt apoptosis.

## Data Availability

Data and materials of this work are available on reasonable request.

## Abbreviations

ABC: Apoptotic Body Count
CD: Celiac Disease
GFD: Gluten Free Diet
IEL: Intraepithelial Lymphocytosis
RCD: Refractory Celiac Disease
